# Genomic Characterization of Dairy Associated *Leuconostoc* Species and Diversity of Leuconostocs in Undefined Mixed Mesophilic Starter Cultures

**DOI:** 10.3389/fmicb.2017.00132

**Published:** 2017-02-03

**Authors:** Cyril A. Frantzen, Witold Kot, Thomas B. Pedersen, Ylva M. Ardö, Jeff R. Broadbent, Horst Neve, Lars H. Hansen, Fabio Dal Bello, Hilde M. Østlie, Hans P. Kleppen, Finn K. Vogensen, Helge Holo

**Affiliations:** ^1^Laboratory of Microbial Gene Technology and Food Microbiology, Department of Chemistry, Biotechnology and Food Science, Norwegian University of Life SciencesÅs, Norway; ^2^Department of Environmental Science, Aarhus UniversityRoskilde, Denmark; ^3^Department of Food Science, University of CopenhagenCopenhagen, Denmark; ^4^Department of Nutrition, Dietetics and Food Sciences, Utah State UniversityLogan, UT, USA; ^5^Department of Microbiology and Biotechnology, Max Rubner-InstitutKiel, Germany; ^6^Sacco SrlCordorago, Italy; ^7^ACD Pharmaceuticals ASLeknes, Norway; ^8^TINE SAOslo, Norway

**Keywords:** dairy, cheese, *leuconostoc*, comparative, genomics, diversity analysis, starter cultures, differentiation

## Abstract

Undefined mesophilic mixed (DL-type) starter cultures are composed of predominantly *Lactococcus lactis* subspecies and 1–10% *Leuconostoc* spp. The composition of the *Leuconostoc* population in the starter culture ultimately affects the characteristics and the quality of the final product. The scientific basis for the taxonomy of dairy relevant leuconostocs can be traced back 50 years, and no documentation on the genomic diversity of leuconostocs in starter cultures exists. We present data on the *Leuconostoc* population in five DL-type starter cultures commonly used by the dairy industry. The analyses were performed using traditional cultivation methods, and further augmented by next-generation DNA sequencing methods. Bacterial counts for starter cultures cultivated on two different media, MRS and MPCA, revealed large differences in the relative abundance of leuconostocs. Most of the leuconostocs in two of the starter cultures were unable to grow on MRS, emphasizing the limitations of culture-based methods and the importance of careful media selection or use of culture independent methods. Pan-genomic analysis of 59 *Leuconostoc* genomes enabled differentiation into twelve robust lineages. The genomic analyses show that the dairy-associated leuconostocs are highly adapted to their environment, characterized by the acquisition of genotype traits, such as the ability to metabolize citrate. In particular, *Leuconostoc mesenteroides* subsp. *cremoris* display telltale signs of a degenerative evolution, likely resulting from a long period of growth in milk in association with lactococci. Great differences in the metabolic potential between *Leuconostoc* species and subspecies were revealed. Using targeted amplicon sequencing, the composition of the *Leuconostoc* population in the five commercial starter cultures was shown to be significantly different. Three of the cultures were dominated by *Ln. mesenteroides* subspecies *cremoris. Leuconostoc pseudomesenteroides* dominated in two of the cultures while *Leuconostoc lactis*, reported to be a major constituent in fermented dairy products, was only present in low amounts in one of the cultures. This is the first in-depth study of *Leuconostoc* genomics and diversity in dairy starter cultures. The results and the techniques presented may be of great value for the dairy industry.

## Introduction

Mesophilic mixed (DL-type) starter cultures used in the production of Dutch-type cheeses are composed of undefined mixtures of homofermentative *Lactococcus lactis* subsp. *lactis* (*Lc. lactis*), *Lactococcus lactis* subsp. *cremoris* (*Lc. cremoris*), *Lactococcus lactis* subsp. *lactis* biovar. *diacetylactis* (*Lc. diacetylactis*) and heterofermentative *Leuconostoc* spp. The latter two provide aroma and texture by metabolizing citrate, producing diacetyl, acetoin and CO_2_, while *Lc. cremoris* and *Lc. lactis* are the major acid producers through fermentation of lactose. In many cheeses, diacetyl is an important aroma compound, and CO_2_ is important for the eye formation (Hugenholtz, 1993). In fermented dairy products, *Leuconostoc* grows in association with the acid-producing lactococci and have been suggested to play a role in promoting the growth of citrate positive *Lactococcus* strains (Vedamuthu, 1994; Bandell et al., 1998; Hache et al., 1999). The importance of *Leuconostoc* in cheese production is widely recognized. DL-type starter cultures are predominantly *Lactococcus* spp., *Leuconostoc* spp. commonly accounting for 1–10% of the starter culture population (Cogan and Jordan, 1994). However, knowledge on the species diversity of *Leuconostoc* included in these starter cultures, or the composition of *Leuconostoc* through the culture production is sparse. Due to the low initial number and relatively weak ability to ferment lactose, *Leuconostoc* spp. are not believed to have a significant effect in the acidification process in the early stages of cheese making (Ardö and Varming, 2010). However, leuconostocs have been shown to dominate the cheese microbiota in the later stages of ripening with added propionic acid bacteria (Porcellato et al., 2013; Østlie et al., 2016). The genus *Leuconostoc* is comprised of 13 species, with the species *Leuconostoc mesenteroides* divided into subspecies *mesenteroides, dextranicum, cremoris*, and *suionicum* (Hemme and Foucaud-Scheunemann, 2004; Gu et al., 2012). The *Leuconostoc* species (or subspecies) relevant for dairy production are *Leuconostoc mesenteroides* subsp. *mesenteroides* (*Ln. mesenteroides*), *Leuconostoc mesenteroides* subsp. *dextranicum* (*Ln. dextranicum*), *Leuconostoc mesenteroides* subsp. *cremoris* (*Ln. cremoris*), *Leuconostoc pseudomesenteroides* (*Ln. pseudomesenteroides*) and *Leuconostoc lactis* (*Ln. lactis*) (Cogan and Jordan, 1994; Thunell, 1995)

The bases for *Leuconostoc* taxonomy are results from cultivation-dependent methods, followed by phenotypic/biochemical characterization or non-specific molecular methods. In addition to being tedious and time-consuming, classical cultivation-dependent methods are known to underestimate the number of *Leuconostoc* spp., especially *Ln. cremoris* (Vogensen et al., 1987; Ward et al., 1990; Auty et al., 2001). In addition, concerns on the lack of stability and reproducibility of phenotypical methods have been raised (Thunell, 1995; Barrangou et al., 2002). Several molecular typing methods, such as RAPD, PFGE, RFLP, Rep-PCR, MLST, MALDI-TOF MS, plasmid profiling and 16S rRNA targeted differentiation have been employed to characterize or identify *Leuconostoc* isolates (Villani et al., 1997; Björkroth et al., 2000; Cibik et al., 2000; Pérez et al., 2002; Sánchez et al., 2005; Vihavainen and Björkroth, 2009; Nieto-Arribas et al., 2010; Alegria et al., 2013; Zeller-Péronnet et al., 2013; Dan et al., 2014; Zhang et al., 2015). However, most of these techniques requiring a preliminary stage of cultivation and comparison of results between the methods and between different laboratories remains challenging. Often, these methods were developed to work with only one or two species of *Leuconostoc*, so they do not provide subspecies differentiation, yield inconclusive results, yield results that are hard to reproduce, or provide arbitrary differentiation of isolates not sufficiently tethered to phenotypic traits. So far, the work by Dr. Ellen Garvie on the growth and metabolism of *Leuconostoc* spp. (Garvie, 1960, 1967, 1969, 1979, 1983; Garvie et al., 1974), and DNA-DNA hybridization studies (Farrow et al., 1989) remains the basis for the taxonomical division of dairy relevant leuconostocs.

The *Leuconostoc* genus has also not been subject to extensive genomic research, and information on the genomic diversity or species population dynamics through the cheese production processes is scarce if available at all. Scientific literature and product information on starter cultures pre-dating the genomic age list *Ln. cremoris* and *Ln. lactis* as the key *Leuconostoc* in undefined mixed mesophilic starter cultures (Lodics and Steenson, 1990; Johansen and Kibenich, 1992; Vedamuthu, 1994). However, in recent years, isolation of *Ln. mesenteroides, Ln. dextranicum*, and *Ln. pseudomesenteroides* is more common from starter cultures or from cheese derivatives (Olsen et al., 2007; Kleppen et al., 2012; Pedersen et al., 2014a,b; Østlie et al., 2016).

Here we present genomic comparative analysis of *Leuconostoc* spp. and present data on the diversity and composition of *Leuconostoc* populations in five commercially available DL-type starter cultures. Using traditional cultivation methods in combination with high-throughput sequencing techniques, we provide robust species and subspecies differentiation, and direct population composition analysis using targeted amplicon-sequencing techniques. To our knowledge, this is the first in-depth genomic work performed on the *Leuconostoc* genus, and the first data published on *Leuconostoc* diversity in DL-type starter cultures.

## Materials and methods

### Cultivation of bacterial strains and starter cultures

All bacterial strains used in this study are listed in Supplementary Table S1. The two different media used for cultivation were de Man Rogosa Sharpe (MRS) (Difco, Detroit, Michigan, USA), and modified PCA (MPCA). PCA (Sigma-Aldrich, Oslo, Norway) was supplemented with 0.5 g/L Tween 80, 5.0 g/L ammonium-citrate, 1 g/L skim milk powder (TINE SA, Oslo, Norway), 0.04 g/L FeSO_4_, 0.2 g/L MgSO_4_, 0.05 g/L MnSO_4_, and 10.0 g/L glucose. Glucose was sterile filtered separately and added after autoclaving. Both media were supplemented with 40 μg/mL vancomycin to select for *Leuconostoc*. Three separate extractions from one batch of each starter cultures (A, B, C, D, and E) were suspended in MPCA to an optical density at 600 nm (OD_600_) of 1.0, serially diluted in 10% (w/v) skim milk and spread plated on MRS and MPCA agar plates in triplicate. The plates were incubated at 22°C for 5 days before colony enumeration. Isolates were transferred to MRS and MPCA broth media, respectively, and cultivated at 22°C for two passages before aliquots were supplemented with 15% (w/v) glycerol (Sigma-Aldrich) and stored at −70°C.

### Genome sequencing, assembly, and annotation

Genomic DNA from *Leuconostoc* isolates was extracted from 1 mL of overnight culture using Qiagen DNeasy Blood & Tissue Kit (Qiagen, Hilden, Germany). The cells were lysed with 40 mg/mL lysozyme (Qiagen, Hilden, Germany) and bead-beating in a FastPrep®-24 (MP Biomedicals, Santa Ana, California) using 0.5 g acid-washed beads (<106 μm) (Sigma-Aldrich) prior to column purification. DNA libraries were made using the Nextera XT DNA Sample Prep kit (Illumina, San Diego, California, USA) according to manufacturer instructions and sequenced with Illumina MiSeq (Illumina, San Diego, California, USA) using V3 chemistry for 33 isolates sequenced at the Norwegian University of Life Sciences, and V2 chemistry for 13 isolates sequenced at the Aarhus University. Raw sequences were adapter trimmed, quality filtered (Q>20), *de novo* assembled using SPAdes V3.7.1 (Nurk et al., 2013) and annotated using the Prokka pipeline (Seemann, 2014). Contigs shorter than 1000 bp or with < 5 times coverage were removed from each assembly prior to gene annotation. Thirteen publicly available genomes of *Leuconostoc* obtained from the National Center for Biotechnology Information (NCBI) database were also included in the dataset (Jung et al., 2012; Meslier et al., 2012; Erkus et al., 2013; Pedersen et al., 2014a,b; Campedelli et al., 2015; Østlie et al., 2016). This whole genome project has been deposited at DDBJ/ENA/GenBank under the BioProject PRJNA352459.

### Genomic analysis

The protein coding sequences of all *Leuconostoc* isolates were compared by an all-against-all approach using BLASTP (Camacho et al., 2009) and grouped into orthologous clusters using GET_HOMOLOGUES (Version 2.0.10) (Contreras-Moreira and Vinuesa, 2013). Pan and core genomes were estimated using the pan-genomic analysis tool PanGP v.1.0.1 (Zhao et al., 2014). Orthologous groups (OGs) were identified via the Markov Cluster Algorithm (MCL) with an inflation value of 1.5 (Enright et al., 2002) and intersected using the compare_clusters.pl script provided with GET_HOMOLOGUES. The orthologous clusters were curated to exclude significantly divergent singletons, which is likely the result of erronous assembly or annotation. A presence/absence matrix for each gene cluster and each genome was constructed for the pan-genome before statistical and clustering analysis of the matrix was performed in R (http://www.r-project.org/). Hierarchal clustering of the pan-genome matrix was performed using complete-linkage UPGMA with Manhattan distances, and a distance cut-off for the number of clusters was determined using the knee of the curve approach (Salvador and Chan, 2004), binning the isolates into genomic lineages. The resulting distance-matrix was used to construct a heatmap with dendrograms using the heatmap.2 function included in the Gplots package (Version 2.16; Warnes et al., 2015) supplemented by the Dendextend package (Version 0.18.3; Galili, 2015).

### Comparative genomics analysis

The genetic potential of individual *Leuconostoc* lineages that were identified by the pan-/core-genome analysis was investigated by producing intra-linage pan-genomes using GET_HOMOLOGUES (Version 2.0.10). The pan-genome for each lineage was analyzed using Blast2GO v4 (Conesa et al., 2005) to identify functionality, and Geneious 8.1.8 (Kearse et al., 2012) to identify sequence variation within orthologous clusters. The lineage pan-genomes were then compared using KEGG databases (Kanehisa and Goto, 2000) and the functional comparative comparison tool found in The SEED Viewer (Overbeek et al., 2014). CRISPR sequences and spacers were identified using the CRISPRFinder tool (Grissa et al., 2007).

### Relative quantification of *Leuconostoc* species in starter cultures

Compositional analysis of *Leuconostoc* in five commercially available starter cultures was performed in triplicates on total DNA isolated from the starter cultures using 1 mL of starter culture diluted to an OD_600_ of 1. The cultures were treated with 20 mg/mL lysozyme (Sigma-Aldrich) and 3U/L mutanolysin (Sigma-Aldrich), mechanically lysed using FastPrep (MP Biomedicals) with 0.5 g of acid-washed beads (<106 μm) (Sigma-Aldrich) and purified using the Qiagen DNeasy Blood & Tissue Kit (Qiagen). A suitable amplicon target was identified by screening the core-genome for nucleotide sequence variation using the sequence alignment metrics functions available in the DECIPHER package v1.16.1 (Wright, 2015). Genes without flanking consensus regions within a 500 bp variable region adequate for differentiation, or did not provide sufficient discrimination from similar sequences in species likely to be present in dairy, were excluded. The locus *eno* encoding for enolase was amplified by PCR using the KAPA HiFi PCR Kit (KAPA Biosystems, Wilmington, Massachusetts, USA) with primers Eno-F (5′-AACACGAAGCTGTTGAATTGCGTG-3′), and Eno-R (5′-GCAAATCCACCTTCATCACCAACTGA-3′). Forward (5′- TCGTCGGCAGCGTCAGATGTGTATAAGAGACAG-) and reverse (5′GTCTCGTGGGCTCGGAGATGTGTATAAGAGACAG-) Illumina adapter overhangs were added to the 5′ end of the primers to allow for Nextera XT DNA indexing of the PCR-products. The resulting libraries were sequenced on an Illumina MiSeq with V3 (2 × 300 bp) reagents. The resulting data were paired-end-joined and quality filtered using PEAR (Zhang et al., 2014) and clustered with a 100% identity level threshold using usearch v7 (Edgar, 2010) with error-minimization from uparse (Edgar, 2013). The resulting sequences were matched against a local BLAST-database produced from the *Leuconostoc* genomes for identification.

## Results

### *Leuconostoc* in dairy starters

**Figure 1:**
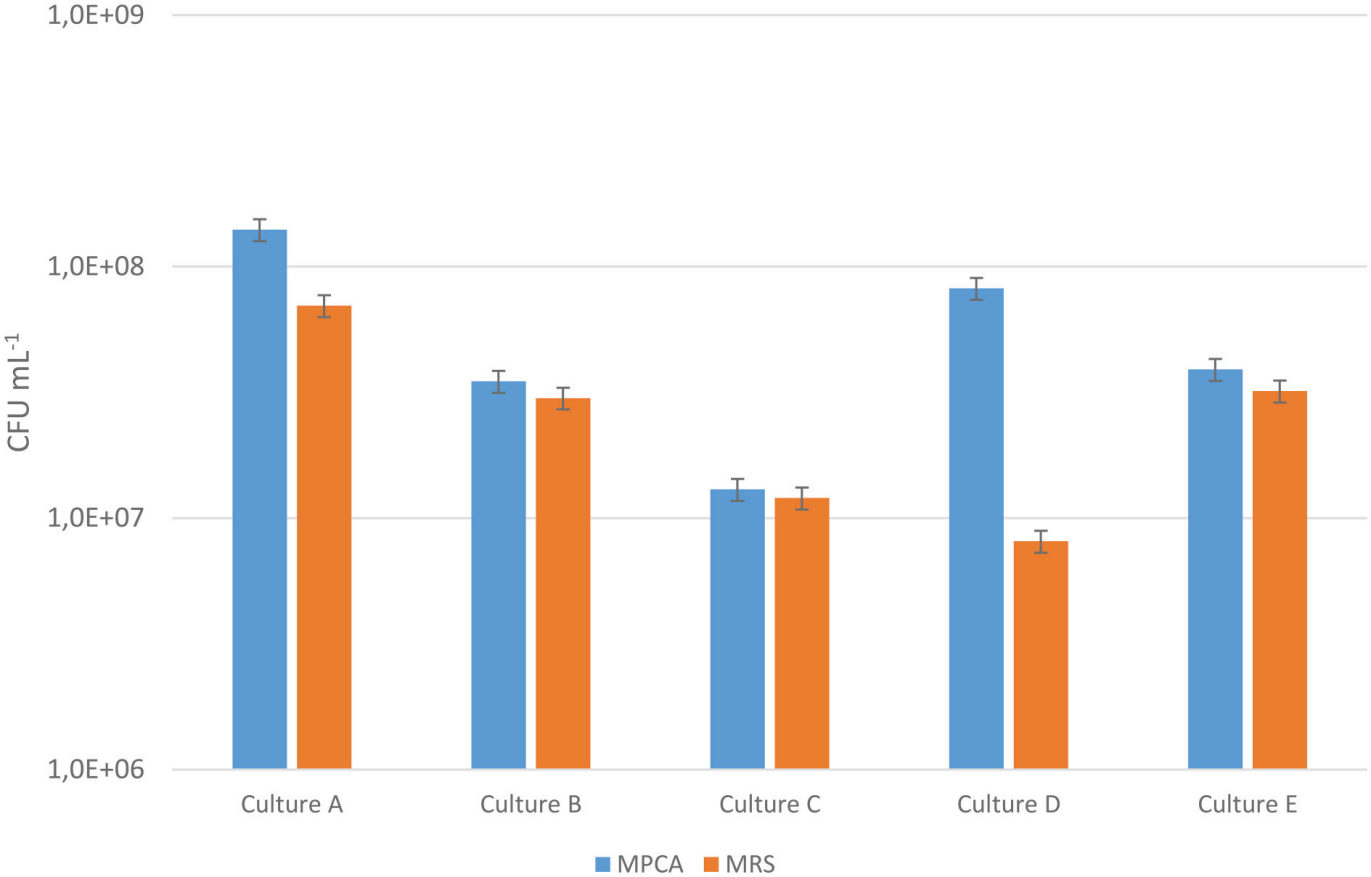
**Bacterial counts for five starter cultures A–E on MRS and MPCA supplemented with vancomycin to select for ***Leuconostoc*****. The counts are the mean of three separate extractions made from the same culture batch and the error bar indicates the standard deviation. The blue bars represent the bacterial counts on MPCA, while the orange bars represent the bacterial counts on MRS. The Y-axis is cut at 1,0E+06 for better readability.

Enumeration on MRS-agar has been reported to underestimate the number of leuconostocs, especially *Ln. cremoris* (Vogensen et al., 1987; Ward et al., 1990; Auty et al., 2001). Bacterial counts were compared in five starter cultures (A, B, C, D, and E) commonly used in the production of Dutch-type cheeses using MRS and MPCA agar with 40 μg/mL vancomycin. The results (Figure 1) showed large differences in the counts between starter cultures for the two media. Cultures A and D gave substantially higher counts on MPCA compared to MRS, while cultures B, C, and E had similar counts on both media. Thus, cultures A and D seemed to contain a large number of *Leuconostoc* strains unable to grow on MRS, while cultures B, C, and E did not.

### Genome sequencing and pan-genomic analysis

**Figure 2:**
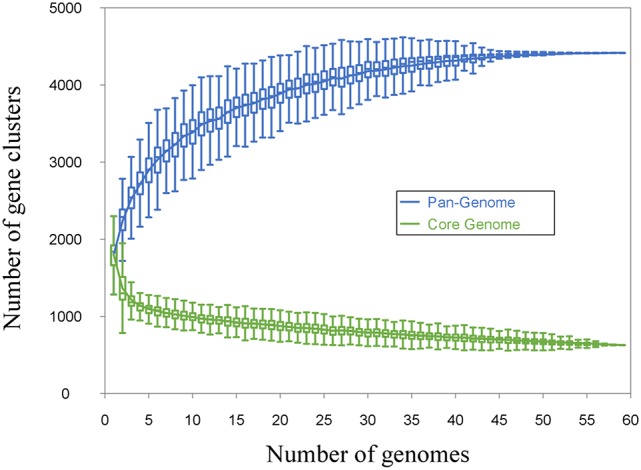
**Pan- and core-genome estimation**. The estimation is made by including genomes one by one, matching the genetic content from each genome, with the growing pan- and the decreasing core-genome. Homologous genes are clustered together in orthologous groups. If the genomes included in the estimation are sufficiently distant from each other with regards to phylogeny, more than one orthologous group can exist for the same gene. The cut-off for this is set by the inflation value in the Markov Cluster Algorithm (MCL), for our dataset the inflation value was set to 1.5. The genetic content was curated for significantly divergent singletons, likely to be the product of erroneous assembly or annotation. The final pan-genome was estimated at 4415 orthologous groups, while the core-genome was estimated at 638 orthologous groups.

**Figure 3:**
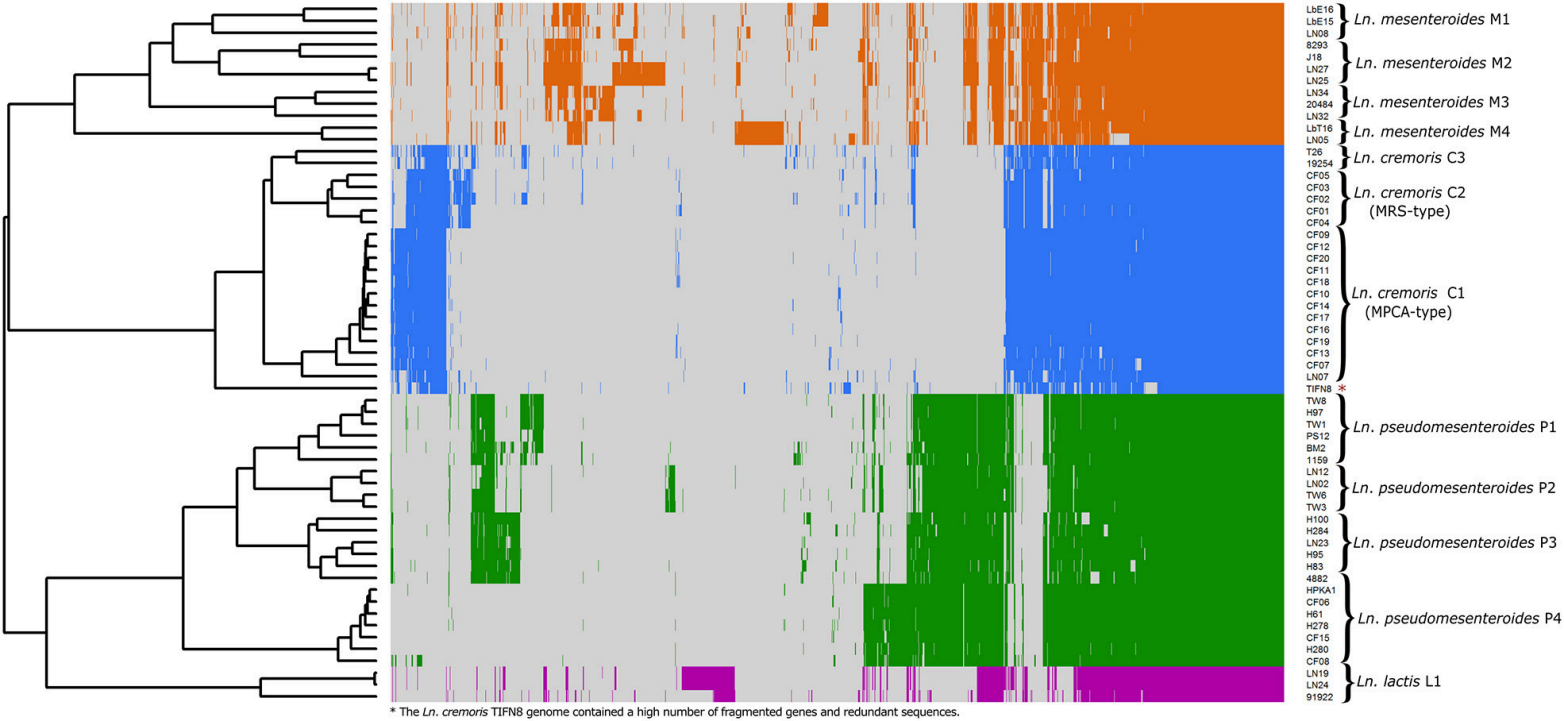
**Differentiation of 59 ***Leuconostoc*** genomes using the pan-genome of 4415 OGs**. Hierarchal clustering of genomes clearly separated *Leuconostoc* species and subspecies. Moreover, the high sensitivity of the method produced twelve robust *Leuconostoc* lineages annotated on the right side of the figure. Four lineages of *Ln. mesenteroides* (colored orange), three lineages of *Ln. cremoris* (colored blue), four lineages of *Ln. pseudomesenteroides* (colored green), and one linage of *Ln. lactis* (colored purple) are shown. (^*^) The *Ln. cremoris* TIFN8 genome was excluded from further analysis because the genome data contained a high number of fragmented genes and redundant sequences. The heatmap was generated with R using the heatmap.2 function included in the Gplots package supplemented by the Dendextend package.

*Leuconostoc* diversity was investigated by whole-genome sequencing of 20 isolates picked from MPCA- and MRS-plates of cultures A and D, and 26 isolates from cheese, including Dutch-type cheese produced using cultures B, C, and E. Lastly, 13 publically available *Leuconostoc* spp. genomes were included in the dataset. All 59 *Leuconostoc* genomes were annotated and the coding sequences (CDS) were compared by a blast-all-against-all approach to identify OGs. Pan- and core-genomes were estimated (Figure 2) using the pan-genomic analysis tool PanGP. After curation, the pan-genome was determined to consist of 4415 OGs, and a core-genome was found to comprise 638 OGs. Differentiation of isolates using hierarchal clustering on the pan-matrix clearly separated *Leuconostoc* species and sub-species (Figure 3). Several of the strains previously identified as *Ln. mesenteroides* subspecies were shown to be *Ln. pseudomesenteroides* by the genomic analysis. Moreover, the NCBI strain LbT16 previously identified as *Ln. cremoris*, was an outlier to the *Ln. cremoris* species branch and was identified in the pan-genomic analysis as *Ln. mesenteroides*. This was further confirmed by alignment of the full-length 16S rRNA, revealing a 100% identity between *Ln. cremoris* LbT16 and *Ln. mesenteroides* type 16S rRNA. Based on sequence similarity and gene content, the pan-genomic clustering divided the 59 leuconostocs into 12 robust *Leuconostoc* lineages across the genus. These included three lineages of *Ln. cremoris* (C1-C3), four lineages of *Ln. pseudomesenteroides* (P1-P4), four lineages of *Ln. mesenteroides* (M1-M4), and one lineage of *Ln. lactis* (L1). The *Ln. cremoris* TIFN8 genome was excluded from further analysis because the genome data contained a high number of fragmented genes and redundant sequences, making it an outlier.

The differences between lineages (Table 1), species and subspecies level (in the case for *Ln. mesenteroides* subsp.) include significantly smaller genomes for *Ln. cremoris* and *Ln. lactis* (1.6–1.8 Mb) compared to *Ln. mesenteroides, Ln. dextranicum*, and *Ln. pseudomesenteroides* (1.8–2.2 Mb). Moreover, the larger genome found in the latter three species contained up to 400 more coding sequences (CDS) than *Ln. cremoris* and *Ln. lactis*. Analysis of functional genomics indicated a closer relationship between *Ln. lactis* and *Ln. pseudomesenteroides*, than that of *Ln. mesenteroides*. Comparison of genetic potential within and between the *Ln. mesenteroides* subspecies showed only minor differences between *Ln. mesenteroides* and *Ln. dextranicum*. Rather, as shown in Figure 3, the variation between the isolates was much greater than the difference between *Ln. mesenteroides* and *Ln. dextranicum*. On the other hand, substantial difference was found between isolates of dairy origin and non-dairy origin. This environment adaptation was also observed for *Ln. lactis*, where *Ln. lactis* 91922, isolated from kimchi was clearly distinguishable from LN19 and LN24 isolated from dairy. Comparison of *Ln. cremoris* and other *Ln. mesenteroides* subspecies isolates revealed that a range of genetic elements found in these species that were missing in *Ln. cremoris*. Apart from some enzymes encoding for rhamnose-containing glucans, *Ln. cremoris* isolates did not have any genetic functionality absent in *Ln. mesenteroides* or *Ln. dextranicum*. Moreover, several truncated genes and deletions were found in *Ln. cremoris* isolates, likely the result of a degenerative evolutionary process through a long period of growth in the milk environment.

**Table 1:** **Average genome size and coding sequences of ***Leuconostoc*** isolates binned into pan-genome lineages**.

**Profile name**	**Average genome size (Mb)**	**Average CDS**
*Ln. cremoris* C1 *(MPCA-type)*	1.680 (±5)	1760 (±20)
*Ln. cremoris* C2 *(MRS-type*)	1.741 (±40)	1822 (±30)
*Ln. cremoris* C3	1.765 (±124)	1956 (±198)
*Ln. mesenteroides* M1	1.869 (±19)	1851 (±7)
*Ln. mesenteroides* M2	2.150 (±123)	2212 (±162)
*Ln. mesenteroides* M3	2.014 (±19)	2074 (±18)
*Ln. mesenteroides* M4	2.061 (±219)	2101 (±173)
*Ln. pseudomesenteroides* P1	2.028 (±47)	2081 (±61)
*Ln. pseudomesenteroides* P2	1.921 (±25)	1925 (±46)
*Ln. pseudomesenteroides* P3	2.063 (±44)	2133 (±60)
*Ln. pseudomesenteroides* P4	2.032 (±61)	2046 (±60)
*Ln. lactis* L1	1.718 (±26)	1700 (±43)

*Information on each individual isolate is included in Supplementary Table S1*.

### Comparative genomics of intra-species *Leuconostoc* lineages

To explore differences in functional genetic potential between the lineages within the species and subspecies, comparative analysis of intra-lineage pan-genomes was performed. The results are included in Supplementary Table S2.

#### (I) *Ln. cremoris* lineages

Comparison of the genetic content for *Ln. cremoris* lineages showed that *Ln. cremoris* C1, C2, and C3 were highly similar and differentiated from each other mostly because of sequence variation in shared OGs. *Ln. cremoris* C1 (MPCA-type), which did not grow on MRS was missing four OGs found in both lineage C2 and C3 (MRS-type). These OGs were annotated *rmlA, rmlB, rmlC*, and *rmlD*, encoding for four enzymes identified in the subsystem “rhamnose containing glycans.” These enzymes are associated with polysaccharide biosynthesis and their presence likely does not explain the inability of C1-type strains to grow on MRS.

#### (II) *Ln. mesenteroides* and *Ln. dextranicum* lineages

Comparison of the genetic content showed a large variance between and within the *Ln. mesenteroides* lineages. Interestingly, no major difference between subspecies *Ln. mesenteroides* and *Ln. dextranicum* was found. *Ln. dextranicum* 20484 is grouped together with *Ln. mesenteroides* isolates LN32 and LN34, while *Ln. dextranicum* LbE16 is grouped together with *Ln. mesenteroides* LbE15 and LN08. This subspecies segregation of *Ln. dextranicum* and *Ln. mesenteroides* was based on the phenotypical ability to produce dextran from sucrose. Dextransucrase, the enzyme involved in this process, is a glucosyltransferase that catalyzes the transfer of glucosyl residues from sucrose to a dextran polymer and releases fructose. Several glucosyltransferases were found within all *Ln. mesenteroides* isolates included in this study, among them several genes encoding for dextransucrases with 40–67% amino acid identity to each other. Genotypically, the potential for dextran production exists within many if not all *Ln. mesenteroides* isolates, and does not differentiate *Ln. mesenteroides* from *Ln. dextranicum*. This finding was manifest by the separation of *Ln. mesenteroides* and *Ln. dextranicum* isolates into four lineages. Functional comparative analyses showed that the presence of the *cit* operon necessary for metabolism of citrate, and the *lacLM* genes is a characteristic of dairy-associated *Ln. mesenteroides, Ln. cremoris* and *Ln. pseudomesenteroides*. In all of the strains in lineages M3 and M4, both the *cit* operon and the *lacLM* genes were present, while strains in lineages M1 and M2 were lacking the *cit* operon, and half of them also lacked the *lacLM* genes. Furthermore, the strains in lineages M1 and M2 contained the genetic potential for metabolism of arabinose, and the two isolates J18 and ATCC8293 also contained genetic potential for xylose and β-glucoside metabolism. The lineage M4 strains LbT16 and LN05 also contained the deletion in the *lacZ* gene which is commonly identified in *Ln. cremoris* type strains. A genetic potential for proteolysis of casein (*prtP*) was identified in *Ln. mesenteroides* lineages M1 and M4, but not in M2 or M3.

#### (III) *Ln. lactis* lineages

The pan-genomic differentiation grouped all the *Ln. lactis* isolates into one lineage. However, differences in genetic potential were found between the kimchi isolate *Ln. lactis* 91922 and dairy isolates LN19 and LN24. *Ln. lactis* 91922 lacked citrate metabolism genes *citCDEFG*, but carried genetic potential for a maltose and glucose specific PTS system, metabolism of arabinose and a CRISPR-Cas operon, that were not found in the other two *Ln. lactis* isolates.

#### (IV) *Ln. pseudomesenteroides* lineages

Despite the significant pan-genomic differences and the sequence variation in shared OGs, the functional differences between lineages of *Ln. pseudomesenteroides* were surprisingly few. *Ln. pseudomesenteroides* P4 was different from the other three lineages with regards to genome synteny and genetic potential. Genetic functionality in the category of methionine biosynthesis, β-glucoside metabolism, sucrose metabolism, as well as an additional lactate dehydrogenase was identified in *Ln. pseudomesenteroides* P4 but not P1, P2, and P3. Moreover, P4 isolates were missing the genes for reduction of diacetyl to acetoin and 2,3-butandiol, and contained genes for a different capsular and extracellular polysaccharide biosynthesis pathway, compared to P1, P2, and P3 isolates.

### Genetic potential of *Leuconostoc*

#### (I) Amino acid biosynthesis

The amino acid requirements of leuconostocs have been described as highly variable between strains. Glutamic acid and valine are required by most leuconostocs, methionine usually stimulates growth, while no *Leuconostoc* are reported to require alanine (Garvie, 1967). Comparative analysis of genes involved in amino acid biosynthesis showed that *Ln. cremoris* and *Ln. mesenteroides* subspecies carried the genetic potential to produce a wide range of amino acids while *Ln. lactis* and *Ln. pseudomesenteroides* did not (Table 2). This included genes encoding biosynthesis of histidine, tryptophan, methionine and lysine. Studies on the amino acid requirement of leuconostocs show that most of the *Ln. mesenteroides* subspecies do require isoleucine and leucine to grow. The *ilv* and *leu* operons involved in biosynthesis of the branched-chain amino acids isoleucine, leucine and valine were present in all *Ln. mesenteroides* isolates, however both operons were truncated when compared to functional *ilv* and *leu* operons from lactococci. The *leuA* gene in the *leuABCD* operon is truncated in leuconostocs (391 aa) compared to lactococci (513 aa) likely resulting in an inactive product and a nonfunctional pathway. This has been documented in the dairy strain *Lactococcus lactis* IL1403 where a similar truncation of the *leuA* gene led to an inactivation of the leucine/valine pathway (Godon et al., 1993). Likewise, the *ilv* operon of sequenced leuconostocs is missing the *ilvD* gene, and has truncated *ilvA* and *ilvH* genes when compared to the lactococcal *ilv* operon. The truncation of *ilvA* has been shown to result in inactivation of the product, and would by itself be sufficient to abort the biosynthesis pathway (Cavin et al., 1999). None of the leuconostocs had genes for biosynthesis of glutamic acid. *Ln. lactis* isolates also lacked the genetic potential for cysteine biosynthesis.

**Table 2:** **Presence of genes encoding enzymes for amino acid biosynthesis**.

**Amino acid pathway**	***Ln. cremoris***	***Ln. mesenteroides***	***Ln. lactis***	***Ln. pseudomesenteroides***
Alanine	+	+	+	+
Arginine	+	+	+	+
Aspartate	+	+	+	+
Cysteine	+	+	−	+
Glutamine	−	−	+	+
Glutamic acid	−	−	−	−
Glycine	+	+	+	+
Histidine	+	+	−	−
Isoleucine	−	−	−	−
Leucine	−	−	−	−
Lysine	+	+	+	−
Methionine	+	+	−	−
Phenylalanine	+	+	+	+
Proline	+	+	+	+
Serine	+	+	+	+
Threonine	+	+	+	+
Tryptophan	+	+	−	−
Tyrosine	+	+	+	+
Valine	−	−	−	−

*+, presence of predicted pathway functionality; −, absence of predicted pathway functionality*.

#### (II) Carbohydrate metabolism

Differences in the genetic potential within and between the *Leuconostoc* species were analyzed by comparing intra-species pan-genomes using Blast2GO and the Seed Viewer. The *Leuconostoc* genus is composed of heterofermentative bacteria that use the phosphoketolase pathway to ferment hexoses. Therefore, it was not surprising to find that none of the isolates contained the gene for phosphofructokinase, a key enzyme in the Embden-Meyerhof pathway. However, a gene encoding fructose-bisphosphate aldolase class II was present in *Ln. lactis* and *Ln. pseudomesenteroides*. This could indicate a potential for synthesis of fructose-1,6-bisphosphate and glyceraldehyde-3-phosphate through fructose-1-phosphate, and hence homofermentative breakdown of fructose in *Ln. lactis* and *Ln. pseudomesenteroides*.

Comparative analysis of genes related to carbohydrate metabolism revealed big differences between the species (Table 3). All leuconostocs in this study encode beta-galactosidase, enabling utilization of lactose. Interestingly, the dairy *Ln. mesenteroides* have two different beta-galactosidases, *lacZ* and the plasmid-encoded *lacLM* (Obst et al., 1995), while the non-dairy isolates only contain *lacZ*. In *Ln. cremoris, lacZ* contains a large central deletion of 1200 bp between positions 740–1940. The *Ln. lactis* isolates only encode beta-galactosidase through *lacZ*, while the *Ln. pseudomesenteroides* isolates only encode beta-galactosidase through *lacLM*. In *Leuconostoc*, lactose is taken up by the lactose-specific transporter LacS, which couples lactose uptake to the secretion of galactose. LacS contains a C-terminal EIIAGlc-like domain and in *S. thermophilus* it has been shown that this domain can be phosphorylated, causing an increased lactose uptake rate (Gunnewijk and Poolman, 2000). All *Leuconostoc* isolates have this gene, but in *Ln. cremoris lacS* is truncated and lacks the C-terminal domain, possibly affecting lactose uptake and hence, growth rate on lactose. Alignment of all *lacS* sequences from this study revealed a close relationship between *Ln. pseudomesenteroides, Ln. lactis*, and *Ln. mesenteroides* isolates of non-dairy origin. In fact, *lacS* of non-dairy associated *Ln. mesenteroides* is more similar to the *lacS* from *Ln. lactis* and *Ln. pseudomesenteroides* (>75% identity) than that of dairy-associated *Ln. mesenteroides* or *Ln. cremoris* (<36% identity). Genes coding for maltose-phosphorylase (*malP*) and sucrose-6-phosphate hydrolase (*scrB*) were found in *Ln. lactis, Ln. pseudomesenteroides* P4, and *Ln. mesenteroides*, but not *Ln. cremoris*. These enzymes are central to the metabolism of maltose and sucrose. Isolates containing *malP* also contained genes *malR* and *malL*, as well as a maltose epimerase. *Ln. lactis* and *Ln. pseudomesenteroides* also contained the *malEFG* gene cluster encoding for an ABC transporter, however the *malEFG* genes were truncated in *Ln. pseudomesenteroides*. Genes encoding for β-glucosidase (*bglA*) enabling utilization of salicin and arbutin was found in all *Ln. pseudomesenteroides* and *Ln. lactis* isolates, as well as in *Ln. mesenteroides* M2 isolates. The *bglA* gene, was found to be present in all *Ln. cremoris* isolates, as well as *Ln. mesenteroides* M1, M3, and M4 isolates, however the gene was truncated and was identified as inactive by the Seed Viewer. A genetic potential for metabolism of trehalose was found, annotated as *treA* in *Ln. mesenteroides* and the *Ln. lactis* of dairy origin, and as *TrePP* in *Ln. pseudomesenteroides* and *Ln. lactis* 91922. Genes encoding for trehalose transport were not found in *Ln. mesenteroides* M3 and M4, indicating that these lineages are not able to metabolize trehalose from the environment. Xylose isomerase (*xylA*) and xylose kinase (*xylB*) genes were found in all *Leuconostoc* isolates, but the genes were heavily truncated in *Ln. cremoris* isolates and *Ln. mesenteroides* M3 and M4 isolates. Isolates with full length *xylA* and *xylB* genes also contained the gene *xylG*, encoding for a xylose transport protein.

**Table 3:** **Genetic potential for metabolism of carbohydrates indicated by the presence or absence of enzymes crucial to metabolism of substrates**.

	***Ln. cremoris***			***Ln. mesenteroides***				***Ln. pseudomesenteroides***				***Ln. lactis***
**Gene(s)**	**C1 (*n* = 13)**	**C2 (*n* = 5)**	**C3 (*n* = 2)**	**M1 (*n* = 3)**	**M2 (*n* = 4)**	**M3 (*n* = 3)**	**M4 (*n* = 2)**	**P1 (*n* = 6)**	**P2 (*n* = 4)**	**P3 (*n* = 5)**	**P4 (*n* = 8)**	**L1 (*n* = 3)**
*araBAD*	−	−	−	+	+	−	−	−	−	−	−	+(33%)
*malP*	−	−	−	#	+	+	−	+	+	+	+	+
*malEFG*	−	−	−	−	−	−	−	#	#	#	#	+
*malX*	−	−	−	−	−	−	−	−	−	−	−	+
*malL*	−	−	−	+	+	+	−	+	+	+	+	+
*malR*	−	−	−	+	+	+	−	+	+	+	+	+
*lacL*	+	+	+	+(66%)	+(50%)	+	+	+	+	+	+	−
*lacM*	+	+	+	+(66%)	+(50%)	+	+	+	+	+	+	−
*lacZ*	#	#	#	+	+	#	#	−	−	−	−	+
*lacS*	#	#	#	+	+	+	+	+	+	+	+	+
*galEKT*	+	+	+	+	+(75%)	+	+	+	+	+	+	+
*manXYZ*	+	+	+	+	+	+	+	+	+	+	+	+
*manA*	+	+	+	+	+	+	+	+	+	+	+	+
*scrB*	−	−	−	+	+	+	+	−	−	−	+	+
*xylABG*	#	#	#	+	+	#	#	+	+	+	+	+
*treA*	−	−	−	+	+	+	+	−	−	−	−	#(66%)
*trePP*	−	−	−	−	−	−	−	+	+	+	+	+(33%)
*bglA*	#	#	#	#	+	#	#	+	+	+	+	+
*fruA*	−	−	−	−	−	−	−	−	−	−	−	+
*levE*	−	−	−	−	+	+	+	+	+	+	+	−
*frk*	#	#	#	+	+	+	+	+	+	+	+	+
*citCDEFGOS*	+	+	+	+	+(50%)	−	+	+	+	+	+	+(66%)
*fba*	−	−	−	−	−	−	−	+	+	+	+	+

+, gene presence. −, gene absence; #, gene present but truncated. Number in parenthesis signifies percentage of isolates where gene was present. All the isolates were able to metabolize glucose and lactose. The number given in parenthesis is given for the percentage of isolates within the lineage with the gene. Genes are abbreviated as follows: araBAD, arabinose metabolism pathway; malP, maltose phosphorylase; malEFG, maltose transport genes; malX, maltose/maltodextrin binding precursor; malL, sucrose-isomaltose; malR, maltose operon regulatory gene; lacL, beta-galactosidase, big subunit; lacM, beta-galactosidase, small subunit; lacZ, beta-galactosidase; lacS, lactose permease; galEKT, galactose metabolism; manXYZ, mannose transport genes; manA, mannose-6-phosphate isomerase; scrB, sucrose-6-phosphate hydrolase; xylABG, xylose isomerase, xylose kinase, xylose transport protein; treA, trehalose-6-phosphate hydrolase; trePP, trehalose-6-phosphate phosphorylase; bglA, beta-D-glucosidase; fruA and levE, fructose PTS; frk, fructokinase; citCDEFGOS, citrate metabolism operon; fba, fructose bisphosphate aldolase

#### (III) Citrate metabolism

All the dairy strains in this study contained the genes necessary for uptake and metabolism of citrate. These genes are found in an operon comprised of *citC* (citrate lyase ligase), *citDEF* (citrate lyase), *citG* (holo-ACP synthase), *citO* (transcriptional regulator) and *citS* (Na+ dependent citrate transporter). A citrate/malate transporter annotated *cimH* was present in *Ln. mesenteroides* subspecies isolates, but was not present in any of the *Ln. lactis* or *Ln. pseudomesenteroides* isolates. In the *Ln. cremoris* and *Ln. pseudomesenteroides* genomes, the *cit* operon is flanked by two IS116/IS110/IS902 family transposases, suggesting it may have been acquired by horizontal gene transfer. In these bacteria, the operon appears to be located on the chromosome, a finding supported by the genome assembly, which organizes the *cit* operon on a contig containing a number of essential genes, and by read coverage analysis that shows a continuous gapless coverage through the contig, with no elevation in read coverage across the *cit* operon. The *citCDEFGOS* operons of *Ln. mesenteroides* and *Ln. lactis*, however, appear to be located on a plasmid, since in all cases they assembled on a contig, which includes a site of replication and not essential genes. The *cit* operon is highly conserved in the *Ln. cremoris* and *Ln. pseudomesenteroides* genomes with >97% DNA sequence identity between all the isolates. The likely to be plasmid-encoded cit operon found in *Ln. mesenteroides* and *Ln. lactis* genomes is also highly conserved between the isolates (>99% identity), however it is significantly different from the chromosomally encoded *cit* operon present in *Ln. cremoris* and *Ln. pseudomesenteroides* (50–65% DNA sequence identity for each gene). None of the strains of non-dairy origin included in this study contained the citrate genes, indicating that the ability to metabolize citrate plays an important role in the successful adaption to the milk environment.

#### (IV) Proteolytic activity

Leuconostocs grow in association with the lactococci in dairy fermentations, and commonly grow poorly in milk without the presence of lactococci. The general explanation for this poor growth is their lack of proteinase activity, making them dependent on small peptides from lactococcal proteinase activity. Screening all the isolates for genes involved in peptide and proteolytic activity revealed a number of differences between the lineages (Table 4). The genes encoding for the OppABCDF system were found in all *Leuconostoc* genomes. However, in *Ln. cremoris* genomes, the *oppA* gene was missing, and the *oppB* gene was severely truncated. A gene encoding for a PII-type serine proteinase (PrtP) best known for its action on caseins was found in all *Ln. pseudomesenteroides* genomes, dairy *Ln. lactis* genomes, *Ln. mesenteroides* M4 and 33% of *Ln. mesenteroides* M1 genomes. All the sequenced *Leuconostoc* strains coded for a range of peptidases and aminotransferases. The *Ln. cremoris* isolates did not contain the *pepN* gene, but had the other general aminopeptidase gene, *pepC*, which was found to be missing from *Ln. lactis* genomes. The *pepX* gene, encoding for the enzyme x-prolyl dipeptidyl aminopeptidase was truncated in *Ln. cremoris* (534 amino acids) compared to the *pepX* of other *Leuconostoc* strains (778–779 amino acids). The *pepA, pepF, pepO, pepQ, pepS*, and *pepT* genes were present in all *Leuconostoc* isolates. Finally, all *Ln. pseudomesenteroides* have the *pepV* gene, encoding β-ala-dipeptidase. This dipeptidase has been shown to cleave dipeptides with an N-terminal β-Ala or D-ala residue, such as carnosine and to a lesser extent, was shown to catalyze removal of N-terminal amino acids from a few distinct tripeptides in *Lactobacillus delbrueckii* subsp. *lactis* (Vongerichten et al., 1994).

**Table 4:** **Genetic potential for proteolytic activity**.

	***Ln. cremoris***			***Ln. mesenteroides***				***Ln. pseudomesenteroides***				***Ln. lactis***
**Gene(s)**	**C1 (*n* = 13)**	**C2 (*n* = 5)**	**C3 (*n* = 2)**	**M1 (*n* = 3)**	**M2 (*n* = 4)**	**M3 (*n* = 3)**	**M4 (*n* = 2)**	**P1 (*n* = 6)**	**P2 (*n* = 4)**	**P3 (*n* = 5)**	**P4 (*n* = 8)**	**L1 (*n* = 3)**
*prtP*	−	−	−	+(33%)	−	−	+	+	+	+	+	+(66%)
*pepA*	+	+	+	+	+	+	+	+	+	+	+	+
*pepC*	+	+	+	+	+	+	+	+	+	+	+	−
*pepF*	+	+	+	+	+	+	+	+	+	+	+	+
*pepN*	−	−	−	+	+	+	+	+	+	+	+	+
*pepO*	+	+	+	+	+	+	+	+	+	+	+	+
*pepQ*	+	+	+	+	+	+	+	+	+	+	+	+
*pepS*	+	+	+	+	+	+	+	+	+	+	+	+
*pepT*	+	+	+	+	+	+	+	+	+	+	+	+
*pepV*	−	−	+	−	−	−	−	+	+	+	+	−
*pepX*	#	#	#	+	+	+	+	+	+	+	+	−
*oppABCDF*	#	#	#	+	+	+	+	+	+	+	+	+

*+, gene presence; −, gene absence; #, gene(s) present but truncated. Number in parenthesis indicates percentage of isolates where gene was present. Genes are abbreviated as follows: prtP, type-II serine proteinase; pepA, glutamyl aminopeptidase; pepC, aminopeptidase C; pepF, oligoendopeptidase; pepN, aminopeptidase N; pepO, neutral endopeptidase; pepS, aminopeptidase; pepT, peptidase T; pepV, beta-ala-xaa dipeptidase; pepX, xaa-pro dipeptidyl-peptidase; oppABCDF, peptide ABC transporter operon*.

### CRISPR-Cas in *Ln. lactis* and *Ln. pseudomesenteroides*

*Ln. lactis* 91922 and all the *Ln. pseudomesenteroides* isolates included in this study contained CRISPR-Cas genes with repeat regions.

### Composition of leuconostocs in starter cultures

**Figure 4:**
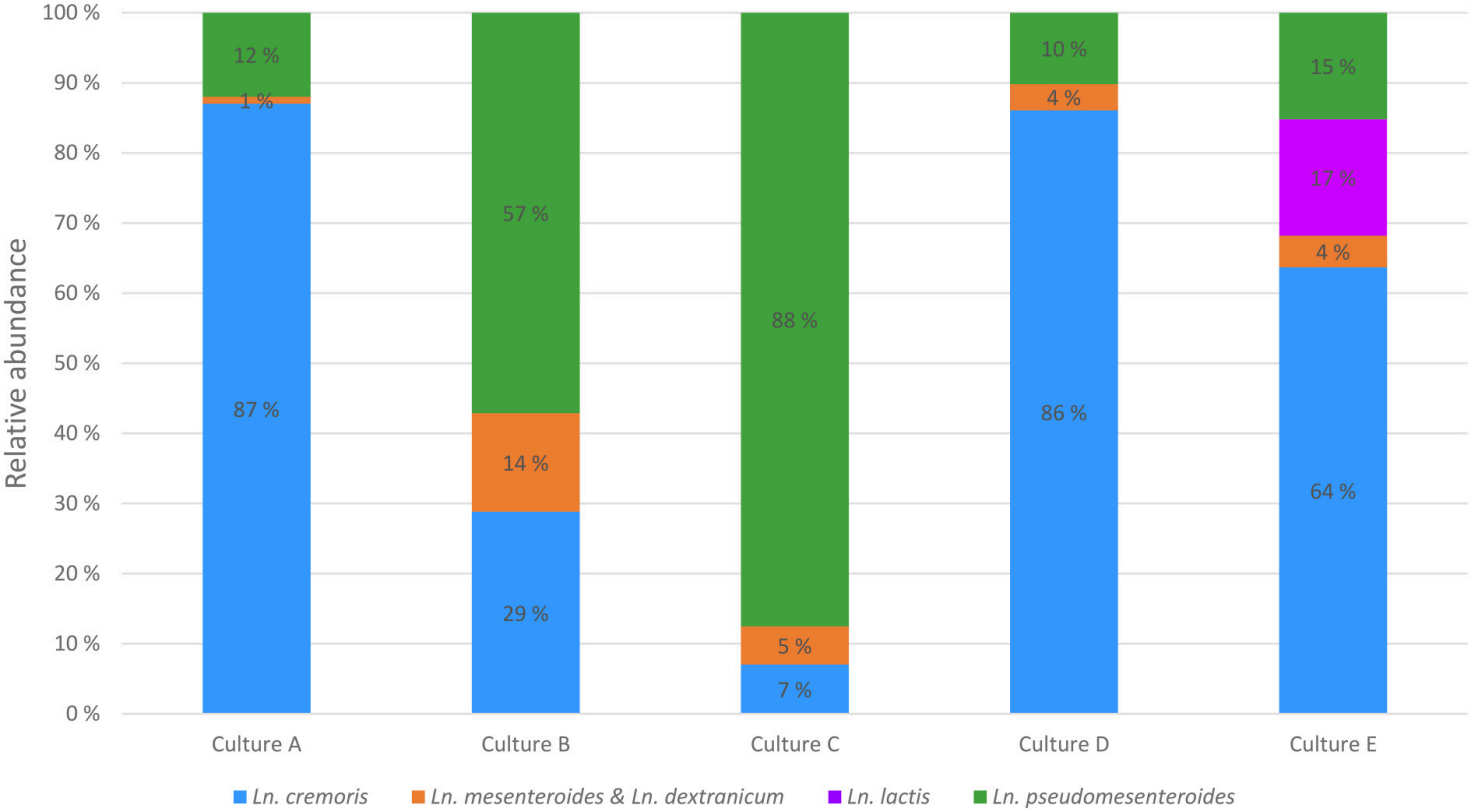
**Composition of leuconostocs in five starter cultures A–E using targeted-amplicon sequencing of the ***eno*** gene**.

The *Leuconostoc* core gene library was used to devise a scheme for species and subspecies quantification in starter cultures by amplicon sequencing. Core genes were screened for sequence variation and for targeted-amplicon suitability. After curation, the top three candidates were 16S rRNA, *rpoB*, and *eno*. While the full-length 16S rRNA sequence enables differentiation of species and subspecies, any region shorter than 500 bp is only able to differentiate between species, and then only when using the nucleotides between position 150–550, encompassing the V2 and V3 regions of 16S rRNA. However, the sequences of 16S rRNA and the *rpoB* loci were too similar to the same genes in lactococci to allow for primer design specific for leuconostocs, and thus were unsuitable for quantification of leuconostocs. The gene encoding enolase (*eno*) did allow for *Leuconostoc* specific primer design, and was used in targeted-amplicon sequencing to analyze the diversity of leuconostocs in the five starter cultures. The analysis revealed great differences between the starter cultures (Figure 4). *Ln. cremoris* dominated the *Leuconostoc* populations in cultures A, D and E, *Ln. pseudomesenteroides* was most abundant in cultures B and C. Most of the *Ln. cremoris* in cultures A and D were of the MPCA type (*Ln. cremoris* C1) unable to grow on MRS, while MRS type *Ln. cremoris* dominated in culture E (data not shown). Relatively low levels of *Ln. mesenteroides* and *Ln. dextranicum* were found in all cultures, the highest being 14% in culture B. *Ln. lactis* was only found in one of the starter cultures, culture E, where it constituted 17% of the leuconostocs.

## Discussion

Decades have passed since Dr. Ellen Garvie laid the foundation for the taxonomy of dairy relevant leuconostocs, and Dr. John Farrow expanded this list to include *Ln. pseudomesenteroides*. Their work has been the basis for classification of leuconostocs since then.

The *Ln. pseudomesenteroides* species was described for the first time in 1898 (Farrow et al., 1989), however its presence in a dairy starter culture was not described before 2014 (Pedersen et al., 2014b). Identification of leuconostocs by phenotypical traits or by partial 16S rRNA sequencing does not reliably distinguish between all species and misidentification has been common. After genomic analysis, several isolates previously identified as *Ln. mesenteroides* subspecies proved to be *Ln. pseudomesenteroides* and isolates may have been misidentified in other studies as well. Surprisingly, the strain LbT16 (Accession. No: LAYV00000000) reported to be *Ln. cremoris* by Campedelli et al. (2015) was identified as *Ln. mesenteroides* when characterized by its genomic content and its full length 16S rRNA sequence. Misidentification of *Ln. cremoris* is also uncommon. Compared to other dairy leuconostocs, *Ln. cremoris* grow slower, to a lower density and not at temperatures of 30°C or higher. In addition, a large proportion of *Ln. cremoris* type strains are not able to grow on MRS. These characteristics provide the means for reliable phenotypical identification of *Ln. cremoris*. However, phenotypical differentiation between other *Ln. mesenteroides* subspecies, *Ln. lactis* and *Ln. pseudomesenteroides* remains unreliable. In this study, dairy relevant leuconostocs are characterized using a genomics approach and the diversity of leuconostocs in five commercial DL-type starter cultures is analyzed.

The genomic analysis clearly separated leuconostocs by species, subspecies, and enabled intra-species differentiation. Interestingly, the genomic analysis did not distinguish *Ln. dextranicum* from *Ln. mesenteroides*. The strain-to-strain variation was higher than the differences between subspecies. The *dextranicum* subspecies has been previously defined by phenotypical traits only and separate subspecies distinction is not justified by the genomic data of this study. On the other hand, the pan-genomic analysis separated *Ln. mesenteroides* isolates by habitat. The dairy strains clearly differ from those isolated from plant material, the former have smaller genomes and utilize a more restricted range of carbohydrates. The two subspecies *Ln. mesenteroides* and *Ln. cremoris* share a large amount of genetic content with high identity scores, reflecting a close phylogenetic relationship. However, many genes present in *Ln. mesenteroides* are found to be truncated, contain deletions or are completely missing in *Ln. cremoris*. Adaptation of dairy strains to the milk environment involved acquisition of the plasmid-encoded *lacLM* by horizontal gene transfer (Obst et al., 1995), which in turn permitted loss of a functional *lacZ*. Some of the dairy *Ln. mesenteroides*, and all of the *Ln. cremoris* isolates carry a deletion in the *lacZ* gene. The dairy *Ln. mesenteroides* and in particular *Ln. cremoris* display telltale signs of a prolonged degenerative evolution, likely the result of a long period of growth in milk. In this environment, the leuconostocs have evolved alongside lactococci. All the dairy strains included in this study contain the *cit* operon comprised of *citC* (citrate lyase ligase), *citDEF* (citrate lyase), *citG* (holo-ACP synthase), *citO* (transcriptional regulator) and *citS* (Na^+^ dependent citrate transporter). The *citCDEFGOS* operon organization is different from the operon in *Lactococcus lactis*, which lacks *citO* and the *citS* transporter (Drider et al., 2004). In citrate positive *Lactococcus lactis*, homologs of *citO* (*citR*) and the *citS* (*citP*) are located on a plasmid (Magni et al., 1994). The presence of the *citCDEFGOS* genes enable so-called citrolactic fermentation, co-metabolism of sugar and citrate providing the cells with higher energy yield and proton motive force (Marty-Teysset et al., 1996). In *Ln. lactis* and *Ln. mesenteroides*, this operon has been linked to a ~22-kb plasmid, inferred by phenotypical studies in combination with monitoring the presence of mobile genetic elements (Lin et al., 1991; Vaughan et al., 1995). In the study by Vaughan et al. (1995), *Ln. mesenteroides* was shown to retain its ability to metabolize citrate after losing three of its four plasmids. Moreover, after curing, a derivative isolate without the ability to degrade citrate still contained the fourth plasmid. Our data indicates that for *Ln. cremoris* and *Ln. pseudomesenteroides*, this is not the case. In all the *Ln. cremoris* and *Ln. pseudomesenteroides* genomes included in this study, the *cit* operon is located on the chromosome in a region with mobile element characteristics. A low level of genetic drift is indicated by the high sequence similarity between the *cit* operons of *Ln. cremoris* and *Ln. pseudomesenteroides* suggesting that the acquisition of these genes is quite recent, possibly from a common donor. The chromosomally encoded *cit* operon of *Ln. cremoris* and *Ln. pseudomesenteroides* was significantly different from the highly conserved and likely to be plasmid-encoded *cit* operon found in *Ln. lactis* and *Ln. mesenteroides*. These results indicate that the plasmid encoded *cit* operon originates from a different source and time. None of the strains of non-dairy origin included in this study contained the citrate metabolism genes, indicating that the ability to metabolize citrate also plays an important role in the successful adaption to the milk environment. The manufacture of Dutch-type cheeses has been going on for centuries and the starter cultures have been maintained by so-called “back slopping” for the last one and a half century, where new milk is inoculated with whey from the previous batch. This technique for propagating starter cultures is still being used and recent studies have shown that the complex starter cultures maintain a highly stable composition with regards to lactococci (Erkus et al., 2013). Culture composition may change over a short period of time depending on growth conditions and bacteriophage predation, but the microbial community is sustained in the long run. In this study, we show a large variation in the amount and composition of the *Leuconostoc* populations in cheeses starter cultures. Three of the starter cultures (A, D, and E) were dominated by *Ln. cremoris*, and for culture A and D, the majority of these were unable to grow on MRS. The other two starter cultures (B and C) were dominated by *Ln. pseudomesenteroides*. Interestingly, the cultures dominated by *Ln. cremoris* also contain *Ln. pseudomesenteroides* strains. *Ln. pseudomesenteroides* growth rates in pure culture are significantly higher than that of *Ln. cremoris* at temperatures above 20°C, so the microbial community is preserved, either by the starter culture developers, or by the microbial community itself. Little knowledge exists on how the diversity of leuconostocs is affected by manufacturing procedures. According to Thunell (1995) and Vedamuthu (1994) the only leuconostocs relevant in dairy are *Ln. cremoris* and *Ln. lactis*, but in this study, *Ln. lactis* was detected only in culture E, which was dominated by *Ln. cremoris*. In two of the starter cultures studies in this work, *Ln. pseudomesenteroides* was the dominating *Leuconostoc*, which shows that they are highly relevant in the production of cheese. This is also reflected by recent studies, where the presence of *Ln. pseudomesenteroides* is more frequently reported (Callon et al., 2004; Porcellato and Skeie, 2016; Østlie et al., 2016). It is tempting to speculate that starter culture manufacturers have altered the conditions for culture propagation or manipulated the strain collections, thereby altering the culture dynamics between strains in favor of *Ln. pseudomesenteroides*.

The differences between the starter cultures could have an impact on the characteristics of the cheese product. *Ln. cremoris* lacks a wide range of genes involved in carbohydrate metabolism and proteolytic activity, and studies have shown that *Ln. cremoris* and *Ln. pseudomesenteroides* differ significantly in their ability to produce a wide range of volatile compounds (Pedersen et al., 2016). Most notably, the amount of acetoin and diacetyl in model-cheeses produced with only *Ln. pseudomesenteroides* was negligible. This was supported by our data, which showed that the *Ln. pseudomesenteroides* P4 isolates lack the genes necessary for reduction of diacetyl to acetoin and 2,3-butandiol. In addition, these isolates lacked the genes *ilvB* and *ilvH* encoding acetolactate synthetase large and small subunits, which is found in all *Ln. mesenteroides* subspecies isolates. However, a different gene *alsS*, encoding the same function, was found in all leuconostocs, including *Ln. pseudomesenteroides*. Studies on α-acetolactate synthase (ALS) and α-acetolactate decarboxylase (ALDC) activity in *Ln. mesenteroides* subspecies and *Ln. lactis* showed that the activity of both ALS and ALDC was higher for *Ln. lactis* (which does not have the *ilv* or *leu* operon) than that of *Ln. cremoris* (which does have part of these two operons) (Monnet et al., 1994). For comparison, the ALS activity of *Lc. lactis* biovar *diacetylactis* was comparable or in some cases even higher than that of *Ln. lactis. Ln. pseudomesenteroides* was not included in the study, but data from semi-hard cheeses comparing the acetoin and diacetyl concentrations revealed lower concentrations in mock starters containing *Ln. pseudomesenteroides* compared to mock starters containing *Ln. cremoris* (Pedersen et al., 2016). This observation could be attributed to the rapid growth rate of *Ln. pseudomesenteroides* when compared to that of *Ln. cremoris*. The presence of the degenerated *ilv* and *leu* operons could somehow be negative to *Ln. cremoris* growth rate. Indeed, when cloning of the *ilv* operon into *Escherichia coli*, the presence of *Leuconostoc ilvB* was strongly detrimental to growth, while recombinant strains with an insertion in the *Leuconostoc ilvB* genes displayed normal growth. Their hypothesis was that expression of *ilvB* without a functional branched chain amino acid biosynthesis mechanism could interfere with energy metabolism via pyruvate (Cavin et al., 1999).

In dairy fermentations, the leuconostocs grow in association with the lactococci. Whether the associative growth is of mutual benefit to the leuconostocs and lactococci has not been determined. Literature often attributes the poor growth of leuconostocs to the lack of protease activity (Vedamuthu, 1994; Thunell, 1995). However, the ability to acidify milk in pure culture has been described for *Ln. pseudomesenteroides* (Cardamone et al., 2011), and we identified genetic potential for caseinolytic activity in *Ln. pseudomesenteroides* in our data. This would enable *Ln. pseudomesenteroides* to grow better in milk than *Ln. cremoris*, which lacks the capacity for protease, as well as a functional peptide uptake system due to the lack of OppA, which is responsible for the uptake of extracellular peptides. An argument for mutually beneficial growth has been made by superimposing metabolic pathways from lactococci and leuconostocs, indicating a potential for metabolic complementation between the two genera (Erkus et al., 2013). One can be forgiven for thinking *Ln. pseudomesenteroides* the better bacteria of the two based on these tidbits of information alone. However, both *Ln. cremoris* and *Ln. pseudomesenteroides* have shown to be significant to the production of cheeses. It is difficult to conclude which *Leuconostoc* species produces the highly subjective matter of the better cheese product. The concentration of volatile compounds, fatty acid derivatives, acetoin, diacetyl, and amino acid derivates in products have been shown to diverge significantly, depending on which *Leuconostoc* species is added to the mixture of lactococci (Pedersen et al., 2016).

In conclusion, the dairy-associated leuconostocs are highly adapted to grow in milk. Comparative genomic analysis reveals great differences between the *Leuconostoc* species and subspecies accustomed to the dairy environment, where they grow in association with the lactococci. The composition of the *Leuconostoc* population is significantly different between commercial starter cultures, which ultimately affects the characteristics and quality of the product. A better understanding of *Leuconostoc* microbial dynamics and the functional role of different dairy leuconostocs could be of great importance and be an applicable tool in ensuring consistent manufacture of high quality product. Currently, no detailed information on the relative amount or diversity of the *Leuconostoc* population in starter cultures is available to the industry. We provide a culture independent method for robust identification and quantification of *Leuconostoc* species in mixed microbial communities, enabling quantification of leuconostocs in starter cultures, as well as monitoring the diversity of leuconostocs through the cheese production process.

## Author contributions

CF isolated and sequenced bacterial strains, performed the sequencing work in Norway (of all CF and H-isolates in addition to all amplicon sequencing), analyzed the data, wrote the R-scripts, devised the methods and wrote the manuscript. FB, HØ, TP, HK, and HN provided bacterial isolates for a larger diversity. WK and LH performed the sequencing of isolates in Denmark. Supervision of danish activities was provided by FV. Supervision of Norwegian activities was provided by HK, HØ, and HH. All co-authors were involved in reviewing and commenting on the manuscript prior to its submission. A large contribution to final editing was made by HN and JB.

## Funding

This work was funded by the Norwegian Research Council, TINE SA, and the Danish Council for Independent Research.

### Conflict of interest statement

The authors declare that the research was conducted in the absence of any commercial or financial relationships that could be construed as a potential conflict of interest.
